# Association of triglyceride-glucose index and derived indices with cataract in middle-aged and elderly Americans: NHANES 2005–2008

**DOI:** 10.1186/s12944-025-02470-4

**Published:** 2025-02-14

**Authors:** Bin Wei, Xin Hu, Ben-Liang Shu, Qin-Yi Huang, Hua Chai, Hao-Yu Yuan, Lin Zhou, Yi-Chong Duan, Li-Li Yao, Zhuo-Er Dong, Xiao-rong Wu

**Affiliations:** The 1st affiliated hospital, Jiangxi Medical College, Nanchang University, Nanchang, Jiangxi China

**Keywords:** TyG, Cataract, Metabolic syndrome

## Abstract

**Aim:**

Explore the relationship between the triglyceride-glucose (TyG) index, along with its derivative indices, and the prevalence of cataracts.

**Methods:**

Data from 20,497 participants in the 2005–2008 National Health and Nutrition Examination Survey (NHANES) were compiled. A final total of 4,499 individuals met the eligibility criteria. Cataract presence was assessed through a self-reported history of cataract surgery. The TyG index and its derivatives—TyG-waist-to-height ratio (WHtR), TyG-neutrophil-to-lymphocyte ratio (NLR), TyG-monocyte-to-lymphocyte ratio (MLR), TyG-log platelet-to-lymphocyte ratio (lgPLR), TyG-log systemic inflammation index (lgSII), and TyG-systemic inflammation response index (SIRI)—were calculated. Statistical analyses included multivariable logistic regression, restricted cubic spline (RCS) curves for nonlinear relationships, and receiver operating characteristic (ROC) analysis.

**Results:**

Higher TyG indices were significantly associated with cataract presence (*P* < 0.001). Specifically, TyG-WHtR, TyG-NLR, TyG-lgPLR, TyG-lgSII, and TyG-SIRI exhibited positive correlations with cataract prevalence, even after adjustment for potential confounders (odds ratio [OR] = 1.17; 95% confidence interval [CI]: 1.01, 1.37; *P* = 0.0403; [OR] = 1.01; 95% [CI]: 1.00, 1.02; *P* = 0.0258; [OR] = 1.08; 95% [CI]: 1.01, 1.16; *P* = 0.0223; [OR] = 1.08; 95% [CI]: 1.03, 1.14; *P* = 0.001; [OR] = 1.02; 95% [CI]: 1.00, 1.04; *P* = 0.0120). Furthermore, the stratified analysis showed that in the 61–85 age group, TyG-lgPLR and TyG-lgSII remained positively associated with cataract prevalence ([OR] = 1.09; 95% [CI]: 1.01, 1.17; *P* = 0.024; [OR] = 1.08; 95% [CI]: 1.02, 1.13; *P* = 0.005). RCS analysis revealed a linear association between these indices and cataracts, with no apparent threshold effect. ROC analysis indicated that TyG-MLR demonstrated the highest predictive ability for cataract presence.

**Conclusion:**

The study results indicate a positive association between TyG-related indicators and cataract the prevalence of cataracts in middle-aged and elderly individuals, suggesting that these markers may serve as practical biomarkers for identifying high-risk individuals. Early detection and management of metabolic and inflammatory factors could contribute to effective preventive strategies for cataract development in the elderly population.

**Supplementary Information:**

The online version contains supplementary material available at 10.1186/s12944-025-02470-4.

## Introduction

Globally, cataract-related vision loss is highly prevalent, especially among the elderly, significantly impacting their quality of life. According to the latest global epidemiological data, cataracts are the primary preventable cause of blindness, with their incidence and associated economic burden rising sharply due to the aging population [1, 2]. The pathophysiological mechanisms of cataracts primarily involve lens protein denaturation and oxidative stress. High blood glucose, metabolic syndrome, smoking, and chronic inflammation are identified as key risk factors [3–5, 6, 7]. Given the high prevalence and detrimental effects of cataracts, investigating emerging metabolic and inflammatory biomarkers related to the condition may aid in early intervention and preventive strategies.

The TyG index, an alternative marker for insulin resistance, has become a critical indicator for assessing metabolic health and has garnered considerable attention in recent years for its predictive value in diabetes and cardiovascular diseases [8–10]. Building on the development of the TyG index, researchers have introduced extended TyG-derived indices. These include TyG-WHtR, TyG-BMI, TyG-NLR, TyG-MLR, TyG-lgPLR, TyG-lgSII, and TyG-SIRI. These indices incorporate factors like obesity (BMI, WHtR) and inflammation (NLR, MLR, etc.) to provide a more comprehensive reflection of metabolic and inflammatory states in the body [11–13]. Although these derivative indices hold significant value in many chronic diseases, their application in ophthalmic diseases, particularly cataracts, remains limited.

Existing studies suggest a close association between cataracts and metabolic abnormalities as well as chronic inflammatory responses [14, 15]. Therefore, combined metabolic and inflammatory markers, such as the TyG index and its derived indices, may influence the onset and progression of cataracts by exacerbating insulin resistance and inflammatory responses. Investigating this potential relationship could offer new insights into the pathogenesis of cataracts and help identify biomarkers for early intervention.

## Methods

### Study population

**Fig. 1:**
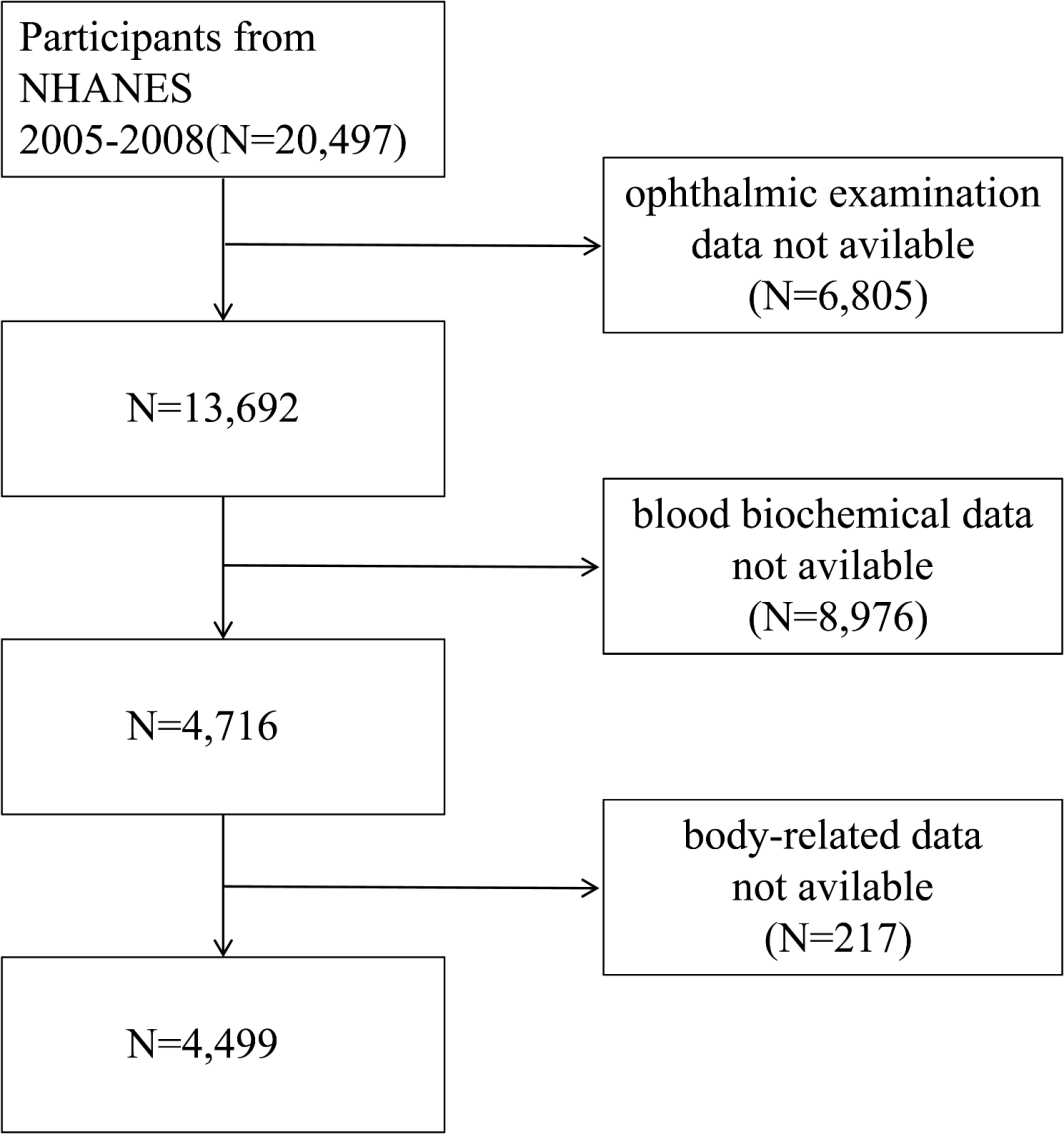
Flow chart of patient screening

A total of 20,497 individuals were included. However, the following data were missing: ophthalmic examination data (6,805 individuals), blood biochemical information (8,976 individuals), and body-related data (217 individuals). Ultimately, 4,499 participants were included (Fig. 1).

### Assessment of cataract

This study references research that used a self-reported history of cataract surgery as the diagnostic criterion. Participants were asked, “Have you ever had a cataract operation?” A positive response to this question was considered indicative of cataract presence. Ambiguous or missing answers were excluded.

### Calculation of TyG-related index

The TyG-related indices are calculated as follows:

$$\begin{array}{l}\:\mathbf{T}\mathbf{y}\mathbf{G}=\mathbf{I}\mathbf{n}\:\left[\mathbf{t}\mathbf{r}\mathbf{i}\mathbf{g}\mathbf{l}\mathbf{y}\mathbf{c}\mathbf{e}\mathbf{r}\mathbf{i}\mathbf{d}\mathbf{e}\right(\mathbf{m}\mathbf{g}/\mathbf{d}\mathbf{L})\\\times\:\:\mathbf{f}\mathbf{a}\mathbf{s}\mathbf{t}\mathbf{i}\mathbf{n}\mathbf{g}\:\mathbf{b}\mathbf{l}\mathbf{o}\mathbf{o}\mathbf{d}\:\mathbf{g}\mathbf{l}\mathbf{u}\mathbf{c}\mathbf{o}\mathbf{s}\mathbf{e}(\mathbf{m}\mathbf{g}/\mathbf{d}\mathbf{L})\:/2]\end{array}$$

$$\:\mathbf{N}\mathbf{L}\mathbf{R}=\:\mathbf{N}\mathbf{e}\mathbf{u}\mathbf{t}\mathbf{r}\mathbf{o}\mathbf{p}\mathbf{h}\mathbf{i}\mathbf{l}\:\mathbf{C}\mathbf{o}\mathbf{u}\mathbf{n}\mathbf{t}/\mathbf{L}\mathbf{y}\mathbf{m}\mathbf{p}\mathbf{h}\mathbf{o}\mathbf{c}\mathbf{y}\mathbf{t}\mathbf{e}\:\mathbf{C}\mathbf{o}\mathbf{u}\mathbf{n}\mathbf{t}$$

$$\:\mathbf{M}\mathbf{L}\mathbf{R}=\:\mathbf{M}\mathbf{o}\mathbf{n}\mathbf{o}\mathbf{c}\mathbf{y}\mathbf{t}\mathbf{e}\:\mathbf{C}\mathbf{o}\mathbf{u}\mathbf{n}\mathbf{t}/\mathbf{L}\mathbf{y}\mathbf{m}\mathbf{p}\mathbf{h}\mathbf{o}\mathbf{c}\mathbf{y}\mathbf{t}\mathbf{e}\:\mathbf{C}\mathbf{o}\mathbf{u}\mathbf{n}\mathbf{t}$$

$$\:\mathbf{I}\mathbf{g}\mathbf{P}\mathbf{L}\mathbf{R}=\:\mathbf{l}\mathbf{g}(\mathbf{P}\mathbf{l}\mathbf{a}\mathbf{t}\mathbf{e}\mathbf{l}\mathbf{e}\mathbf{t}\:\mathbf{C}\mathbf{o}\mathbf{u}\mathbf{n}\mathbf{t}/\mathbf{L}\mathbf{y}\mathbf{m}\mathbf{p}\mathbf{h}\mathbf{o}\mathbf{c}\mathbf{y}\mathbf{t}\mathbf{e}\:\mathbf{C}\mathbf{o}\mathbf{u}\mathbf{n}\mathbf{t})$$

$$\begin{array}{l}\:\mathbf{I}\mathbf{g}\mathbf{S}\mathbf{I}\mathbf{I}\:=\mathbf{l}\mathbf{g}(\mathbf{N}\mathbf{e}\mathbf{u}\mathbf{t}\mathbf{r}\mathbf{o}\mathbf{p}\mathbf{h}\mathbf{i}\mathbf{l}\:\mathbf{C}\mathbf{o}\mathbf{u}\mathbf{n}\mathbf{t}\:\\\times\:\:\mathbf{P}\mathbf{l}\mathbf{a}\mathbf{t}\mathbf{e}\mathbf{l}\mathbf{e}\mathbf{t}\:\mathbf{C}\mathbf{o}\mathbf{u}\mathbf{n}\mathbf{t}/\mathbf{L}\mathbf{y}\mathbf{m}\mathbf{p}\mathbf{h}\mathbf{o}\mathbf{c}\mathbf{y}\mathbf{t}\mathbf{e}\:\mathbf{C}\mathbf{o}\mathbf{u}\mathbf{n}\mathbf{t})\end{array}$$

$$\begin{array}{l}\:\mathbf{S}\mathbf{I}\mathbf{R}\mathbf{I}=\:\mathbf{N}\mathbf{e}\mathbf{u}\mathbf{t}\mathbf{r}\mathbf{o}\mathbf{p}\mathbf{h}\mathbf{i}\mathbf{l}\:\mathbf{C}\mathbf{o}\mathbf{u}\mathbf{n}\mathbf{t}\:\\\times\:\:\mathbf{M}\mathbf{o}\mathbf{n}\mathbf{o}\mathbf{c}\mathbf{y}\mathbf{t}\mathbf{e}\:\mathbf{C}\mathbf{o}\mathbf{u}\mathbf{n}\mathbf{t}/\mathbf{L}\mathbf{y}\mathbf{m}\mathbf{p}\mathbf{h}\mathbf{o}\mathbf{c}\mathbf{y}\mathbf{t}\mathbf{e}\:\mathbf{C}\mathbf{o}\mathbf{u}\mathbf{n}\mathbf{t}\end{array}$$

$$\:\mathbf{B}\mathbf{M}\mathbf{I}=\mathbf{H}\mathbf{e}\mathbf{i}\mathbf{g}\mathbf{h}\mathbf{t}\left(\mathbf{m}\right)2$$

$$\:\mathbf{W}\mathbf{H}\mathbf{t}\mathbf{R}=\left(\mathbf{c}\mathbf{m}\right)/\:\mathbf{H}\mathbf{e}\mathbf{i}\mathbf{g}\mathbf{h}\mathbf{t}\left(\mathbf{c}\mathbf{m}\right)$$

$$\:\mathbf{T}\mathbf{y}\mathbf{G}-\mathbf{N}\mathbf{L}\mathbf{R}=\mathbf{T}\mathbf{y}\mathbf{G}\times\:\mathbf{N}\mathbf{L}\mathbf{R}$$

$$\:\mathbf{T}\mathbf{y}\mathbf{G}-\mathbf{M}\mathbf{L}\mathbf{R}=\mathbf{T}\mathbf{y}\mathbf{G}\times\:\mathbf{M}\mathbf{L}\mathbf{R}$$

$$\:\mathbf{T}\mathbf{y}\mathbf{G}-\mathbf{l}\mathbf{g}\mathbf{P}\mathbf{L}\mathbf{R}=\mathbf{T}\mathbf{y}\mathbf{G}\times\:\mathbf{l}\mathbf{g}\mathbf{P}\mathbf{L}\mathbf{R}$$

$$\:\mathbf{T}\mathbf{y}\mathbf{G}-\mathbf{l}\mathbf{g}\mathbf{S}\mathbf{I}\mathbf{I}=\mathbf{T}\mathbf{y}\mathbf{G}\times\:\mathbf{l}\mathbf{g}\mathbf{S}\mathbf{I}\mathbf{I}$$

$$\:\mathbf{T}\mathbf{y}\mathbf{G}-\mathbf{S}\mathbf{I}\mathbf{R}\mathbf{I}=\mathbf{T}\mathbf{y}\mathbf{G}\times\:\mathbf{S}\mathbf{I}\mathbf{R}\mathbf{I}$$

$$\:\mathbf{T}\mathbf{y}\mathbf{G}-\mathbf{B}\mathbf{M}\mathbf{I}=\mathbf{T}\mathbf{y}\mathbf{G}\times\:\mathbf{B}\mathbf{M}\mathbf{I}$$

$$\:\mathbf{T}\mathbf{y}\mathbf{G}-\mathbf{W}\mathbf{H}\mathbf{t}\mathbf{R}=\mathbf{T}\mathbf{y}\mathbf{G}\times\:\mathbf{W}\mathbf{H}\mathbf{t}\mathbf{R}$$

### Covariates

The variables included in this study encompass general demographic data, physical examination findings, and laboratory data, specifically age, race, gender, marital status, education level, BMI, waist circumference, triglycerides (TG), lymphocyte count(LY), and total cholesterol (TC). Additionally, self-reported daily health information, such as smoking, diabetes, and alcohol consumption status, is also included.

### Statistical analysis

Statistical analysis and plotting were performed using EmpowerStats (2.0) and R (4.1.1). Given that NHANES employs a detailed, multi-stage probability sampling method, all analyses incorporated appropriate weights to account for the complexities of the survey design. The normality of continuous variables was assessed using the Shapiro-Wilk test. Variables following a normal distribution were analyzed using the Student’s t-test, while those with non-normal distributions were assessed using the Mann-Whitney U test. Weighted chi-square tests were conducted to evaluate the baseline characteristics of the populations. Multivariable logistic regression was used to assess the relationship between continuous exposure variables and binary outcome variables. The Box-Tidwell test was employed to examine the linearity of the relationship between continuous predictor variables and the log odds of the outcome variable. Multicollinearity was assessed by calculating the variance inflation factor (VIF), with a threshold of VIF ≥ 5 established as indicative of significant multicollinearity. If multicollinearity was detected, redundant variables were removed. Results are presented as adjusted odds ratios (OR) with 95% confidence intervals (CI). Three models were developed: Model 1, with no adjustments for covariates; Model 2, adjusted for age, gender, and race; and Model 3, adjusted for age, gender, race, marital status, education level, income-to-poverty ratio, spouse’s education level, hypertension, alcohol consumption, red blood cell count, high-density lipoprotein cholesterol, and total cholesterol. After adjusting for the covariates in Model 3, RCS regression was performed using the 25th, 50th, and 75th percentiles of the TYG index to assess the non-linear relationship between the TYG index and cataracts. Additionally, a piecewise linear regression model was employed to evaluate the potential non-linear association between cataracts and the TYG index, identify threshold effects, and calculate the inflection points. We also utilized ROC curves to calculate and compare the areas under the curve (AUC) to assess diagnostic performance. Finally, subgroup analyses and interaction tests were conducted based on gender, age (< 40, 40–60, > 60 years), race, education level, spouse’s education level, marital status, smoking, alcohol consumption, hypertension, and diabetes to explore potential differences across various populations (*P* < 0.05 was considered statistically significant).

## Results

### Basic characteristics

Among the 4,499 participants, 49.14% were male. The overall prevalence of cataracts was 10.1%(Table 1). Compared to the normal group, the TyG-related indices in the cataract group were higher, except for TyG-BMI (*P* < 0.001). In addition, patients in the cataract group exhibited significantly higher levels of age, HDL-C, monocytes, neutrophils, fasting blood glucose, and TG, while PIR, LY, RBC, HB, PLT, and TC levels were significantly lower (*P* < 0.050).

**Table 1:** Based on the baseline characteristics of the study population ascertained by NHANES from 2005 to 2008

Characteristics	Total (*N* = 4499)	Non-cataract (*N* = 4088)	Cataract (*N* = 411)	*P*-value
Age (years)	49.37 ± 18.12	46.86 ± 16.82	74.30 ± 9.84	**< 0.001**
≤ 41	1647 (36.61%)	1641 (40.14%)	6 (1.46%)	**< 0.001**
41–60	1450 (32.23%)	1424 (34.83%)	26 (6.33%)	
≥ 61	1402 (31.16%)	1023 (25.02%)	379 (92.21%)	
Poverty-to-income ratio	2.63 ± 1.54	2.66 ± 1.56	2.37 ± 1.32	**0.003**
Body mass index (kg/m2)	28.74 ± 6.45	28.79 ± 6.49	28.24 ± 5.96	0.180
Lymphocyte	2.04 ± 0.91	2.05 ± 0.78	1.94 ± 1.75	**< 0.001**
Monocyte	0.54 ± 0.19	0.53 ± 0.18	0.60 ± 0.27	**< 0.001**
Neutrophil	4.09 ± 1.69	4.07 ± 1.69	4.31 ± 1.68	**< 0.001**
Erythrocyte	4.73 ± 0.52	4.75 ± 0.51	4.50 ± 0.53	**< 0.001**
Hemoglobin	14.33 ± 1.62	14.38 ± 1.61	13.84 ± 1.59	**< 0.001**
Platelet	267.62 ± 70.72	268.97 ± 70.54	254.13 ± 71.15	**< 0.001**
Fasting Blood Glucose	108.31 ± 37.12	107.48 ± 37.21	116.60 ± 35.17	**< 0.001**
High-Density Lipoprotein	54.78 ± 16.29	54.63 ± 16.29	56.23 ± 16.22	**0.047**
Total Cholesterol	197.17 ± 42.52	197.46 ± 42.49	194.28 ± 42.72	0.117
Triglyceride	139.97 ± 106.77	139.74 ± 109.29	142.31 ± 77.30	**< 0.001**
Gender (%)				**0.030**
Male	2211 (49.14%)	2030 (49.66%)	181 (44.04%)	
Female	2288 (50.86%)	2058 (50.34%)	230 (55.96%)	
Race(%)				**< 0.001**
Mexican American	829 (18.43%)	799 (19.55%)	30 (7.30%)	
Other Hispanic	348 (7.74%)	319 (7.80%)	29 (7.06%)	
Non-Hispanic White	2213 (49.19%)	1914 (46.82%)	299 (72.75%)	
Non-Hispanic Black	922 (20.49%)	876 (21.43%)	46 (11.19%)	
Other Race	187 (4.16%)	180 (4.40%)	7 (1.70%)	
Education Level(%)				**< 0.001**
Less Than 9th Grade	543 (12.07%)	463 (11.33%)	80 (19.46%)	
9-11th Grade	737 (16.38%)	669 (16.36%)	68 (16.55%)	
High School Grad	1106 (24.58%)	985 (24.09%)	121 (29.44%)	
Some College or AA degree	1205 (26.78%)	1114 (27.25%)	91 (22.14%)	
College Graduate or above	908 (20.18%)	857 (20.96%)	51 (12.41%)	
Marital status(%)				**< 0.001**
Married	2458 (54.63%)	2251 (55.06%)	207 (50.36%)	
Widowed	366 (8.14%)	229 (5.60%)	137 (33.33%)	
Divorced	471 (10.47%)	428 (10.47%)	43 (10.46%)	
Separated	153 (3.40%)	146 (3.57%)	7 (1.70%)	
Never married	690 (15.34%)	682 (16.68%)	8 (1.95%)	
Living with partner	361 (8.02%)	352 (8.61%)	9 (2.19%)	
Person’s Spouse Education Level				**< 0.001**
Less Than 9th Grade	294 (6.53%)	262 (6.41%)	32 (7.79%)	
9-11th Grade	307 (6.82%)	283 (6.92%)	24 (5.84%)	
High School Grad	594 (13.20%)	522 (12.77%)	72 (17.52%)	
Some College or AA degree	598 (13.29%)	553 (13.53%)	45 (10.95%)	
College Graduate or above	577 (12.83%)	549 (13.43%)	28 (6.81%)	
Less Than 9th Grade	2129 (47.32%)	1919 (46.94%)	210 (51.09%)	
Alcohol drinking(%)				**< 0.001**
YES	3008 (66.86%)	2773 (67.83%)	235 (57.18%)	
NO	1267 (28.16%)	1106 (27.05%)	161 (39.17%)	
Other	224 (4.98%)	209 (5.11%)	15 (3.65%)	
Hypertension(%)				**< 0.001**
YES	1551 (34.47%)	1304 (31.90%)	247 (60.10%)	
NO	2948 (65.53%)	2784 (68.10%)	164 (39.90%)	
diabetes				**< 0.001**
YES	512 (11.38%)	404 (9.88%)	108 (26.28%)	
NO	3904 (86.77%)	3615 (88.43%)	289 (70.32%)	
Other	83 (1.84%)	69 (1.69%)	14 (3.41%)	
Smoking status(%)				**0.001**
YES	2189 (48.66%)	1958 (47.90%)	231 (56.20%)	
NO	2310 (51.34%)	2130 (52.10%)	180 (43.80%)	
TYG	8.72 ± 0.67	8.70 ± 0.68	8.87 ± 0.58	**< 0.001**
TYG-WHtR	5.16 ± 1.03	5.13 ± 1.03	5.48 ± 0.94	**< 0.001**
TYG-BMI	251.76 ± 64.53	251.80 ± 64.95	251.38 ± 60.26	0.755
TYG-NLR	19.13 ± 10.12	18.74 ± 9.81	23.02 ± 12.18	**< 0.001**
TYG-MLR	2.49 ± 1.11	2.42 ± 1.04	3.15 ± 1.50	**< 0.001**
TYG-lgPLR	18.53 ± 1.89	18.48 ± 1.87	19.02 ± 1.98	**< 0.001**
TYG-lgSII	23.58 ± 2.84	23.50 ± 2.83	24.39 ± 2.89	**< 0.001**
TYG-SIRI	10.53 ± 7.48	10.21 ± 7.19	13.76 ± 9.37	**< 0.001**

Note: All values are presented as proportion (%) or mean (standard error); to analyze the continuous variables, a weighted Student’s t-test was employed, while for categorical variables, a chi-square test was utilized. Significant values are in [bold]. Abbreviations: TyG, triglyceride-glucose index; WHtR, body mass index; BMI, Body Mass Index; NLR, Neutrophil-to-Lymphocyte Ratio; MLR, Monocyte-to-Lymphocyte Ratio; lgPLR, Log-Transformed Platelet-to-Lymphocyte Ratio; lgSII, Log-Transformed Systemic Immune-Inflammation Index; SIRI, Systemic Inflammation Response Index

### Association between cataract and TyG-related indices

Table 2 analyzes the association between TyG-related indices and cataracts using multivariable regression models. In Model 1, all TyG-related indices except TyG-BMI showed a significant positive correlation with cataract presence. This positive association for TyG-WHtR, TyG-NLR, TyG-lgPLR, TyG-lgSII, and TyG-SIRI persisted even in the fully adjusted model (OR = 1.17, 95% CI: 1.01, 1.37, *P* = 0.04; OR = 1.01, 95% CI: 1.00, 1.02, *P* = 0.03; OR = 1.08, 95% CI: 1.01, 1.16, *P* = 0.02; OR = 1.08, 95% CI: 1.03, 1.14, *P* = 0.001; OR = 1.02, 95% CI: 1.00, 1.04, *P* = 0.01). This indicates that for each unit increase in TyG-WHtR, TyG-NLR, TyG-lgPLR, TyG-lgSII, and TyG-SIRI, the likelihood of cataract prevalence increased by 17%, 2%, 8%, 8%, and 2%, respectively (*P* < 0.05). The stratified logistic regression analysis showed that the association between TyG-related indicators and cataract risk varied by age group. Stronger correlations were observed in middle-aged and elderly populations.

**Table 2:** Multivariable logistic regression models for the association between TyG-related index and cataract in adults in the NHANES 2005–2008

Exposure	Crude Model (Model 1)	Partially Adjusted Model (Model 2)	Fully Adjusted Model (Model 3)
	OR (95% CI) *P*-value	OR (95% CI) *P*-value	OR (95% CI) *P*-value
Age: 20–85			
TYG	1.42 (1.23, 1.64) **< 0.001**	1.22 (0.99, 1.50) 0.058	1.28 (0.99, 1.67) 0.064
TYG-WHtR	1.37 (1.25, 1.51) **< 0.001**	1.18 (1.04, 1.35) **0.013**	1.17 (1.01, 1.37) **0.040**
TYG-BMI	1.00 (1.00, 1.00) 0.900	1.00 (1.00, 1.00) 0.066	1.00 (1.00, 1.00) 0.171
TYG-NLR	1.03 (1.02, 1.04) **< 0.001**	1.01 (1.00, 1.03) **0.013**	1.01 (1.00, 1.02) **0.026**
TYG-MLR	1.54 (1.42, 1.66) **< 0.001**	1.10 (1.00, 1.21) **0.049**	1.09 (0.99, 1.20) 0.073
TYG-lgPLR	1.17 (1.11, 1.23) **< 0.001**	1.07 (1.00, 1.14) **0.046**	1.08 (1.01, 1.16) **0.022**
TYG-lgSII	1.12 (1.08, 1.16) **< 0.001**	1.07 (1.03, 1.12) **0.002**	1.08 (1.03, 1.14) **0.001**
TYG-SIRI	1.05 (1.04, 1.06) **< 0.001**	1.02 (1.01, 1.04) **0.007**	1.02 (1.00, 1.04) **0.012**
Age: 20–40			
TYG	1.91 (0.72, 5.07) 0.196	2.00 (0.82, 4.90) 0.128	1.98 (0.88, 5.78) 0.060
TYG.WHTR	1.31 (0.65, 2.63) 0.447	1.39 (0.70, 2.79) 0.350	1.44 (0.59, 3.52) 0.424
TYG.BMI	1.01 (1.00, 1.02) 0.245	1.01 (1.00, 1.02) 0.240	1.01 (1.00, 1.02) 0.216
TYG.NLR	1.02 (0.96, 1.09) 0.554	1.03 (0.97, 1.10) 0.356	1.07 (0.96, 1.19) 0.204
TYG.MLR	0.86 (0.33, 2.23) 0.757	0.93 (0.35, 2.46) 0.878	1.03 (0.39, 2.72) 0.947
TYG.LGPLR	1.19 (0.77, 1.84) 0.426	1.23 (0.81, 1.88) 0.329	1.55 (0.80, 2.98) 0.190
TYG.LGSII	1.23 (0.95, 1.59) 0.112	1.28 (0.99, 1.66) 0.061	1.79 (1.04, 3.09) **0.036**
TYG.SIRI	1.02 (0.95, 1.11) 0.536	1.04 (0.96, 1.12) 0.376	1.04 (0.94, 1.14) 0.466
Age: 41–60			
TYG	1.13 (0.65, 1.96) 0.655	0.99 (0.55, 1.81) 0.985	1.08 (0.51, 2.30) 0.839
TYG.WHTR	0.99 (0.67, 1.45) 0.941	0.89 (0.59, 1.35) 0.587	0.83 (0.50, 1.36) 0.457
TYG.BMI	1.00 (0.99, 1.00) 0.547	1.00 (0.99, 1.00) 0.456	1.27 (1.26, 1.28) **< 0.001**
TYG.NLR	1.02 (1.00, 1.05) **0.040**	1.03 (1.00, 1.06) **0.024**	1.05 (1.01, 1.10) **0.019**
TYG.MLR	1.46 (1.05, 2.03) **0.023**	1.32 (0.91, 1.90) 0.141	1.43 (0.96, 2.12) 0.078
TYG.LGPLR	1.03 (0.83, 1.28) 0.770	0.99 (0.78, 1.25) 0.920	1.03 (0.80, 1.32) 0.809
TYG.LGSII	1.10 (0.96, 1.26) 0.186	1.09 (0.94, 1.27) 0.266	inf. (inf., inf.) **< 0.001**
TYG.SIRI	1.05 (1.01, 1.10) **0.007**	1.05 (1.01, 1.09) **0.021**	1.07 (1.02, 1.13) **0.010**
Age: 61–85			
TYG	1.07 (0.88, 1.30) 0.490	1.26 (1.00, 1.59) 0.051	1.30 (0.97, 1.75) 0.079
TYG.WHTR	1.07 (0.94, 1.22) 0.281	1.26 (1.09, 1.46) **0.002**	1.29 (1.08, 1.53) **0.004**
TYG.BMI	1.00 (1.00, 1.00) 0.146	1.00 (1.00, 1.01) **0.014**	1.00 (1.00, 1.01) **0.032**
TYG.NLR	1.03 (1.02, 1.04) **< 0.001**	1.01 (1.00, 1.02) 0.078	1.01 (1.00, 1.02) 0.106
TYG.MLR	1.25 (1.15, 1.37) **< 0.001**	1.08 (0.98, 1.19) 0.114	1.07 (0.97, 1.19) 0.168
TYG.LGPLR	1.07 (1.01, 1.14) **0.022**	1.08 (1.00, 1.15) **0.036**	1.09 (1.01, 1.17) **0.024**
TYG.LGSII	1.08 (1.04, 1.13) **< 0.001**	1.07 (1.02, 1.12) **0.008**	1.08 (1.02, 1.13) **0.005**
TYG.SIRI	1.04 (1.02, 1.05) **< 0.001**	1.02 (1.00, 1.03) 0.064	1.02 (1.00, 1.03) 0.079

Model 1, no covariates were adjusted. Model 2, age, gender, and race were adjusted. Model 3, age, gender, race, marital status, education level, income-to-poverty ratio, person’s spouse education level, hypertension, alcohol, erythrocyte, high-density lipoprotein, and total cholesterol were adjusted. All values are based on multivariate logistic regression analysis adjusting for [relevant confounders such as age, gender, etc.]. Significant values are in [bold]. Abbreviations: OR, odds ratio; 95% CI, 95% confidence interval; TyG, triglyceride-glucose index; WHtR, body mass index; BMI, Body Mass Index; NLR, Neutrophil-to-Lymphocyte Ratio; MLR, Monocyte-to-Lymphocyte Ratio; lgPLR, Log-Transformed Platelet-to-Lymphocyte Ratio; lgSII, Log-Transformed Systemic Immune-Inflammation Index; SIRI, Systemic Inflammation Response Index

### RCS curve plotting and threshold effect analysis

**Fig. 2:**
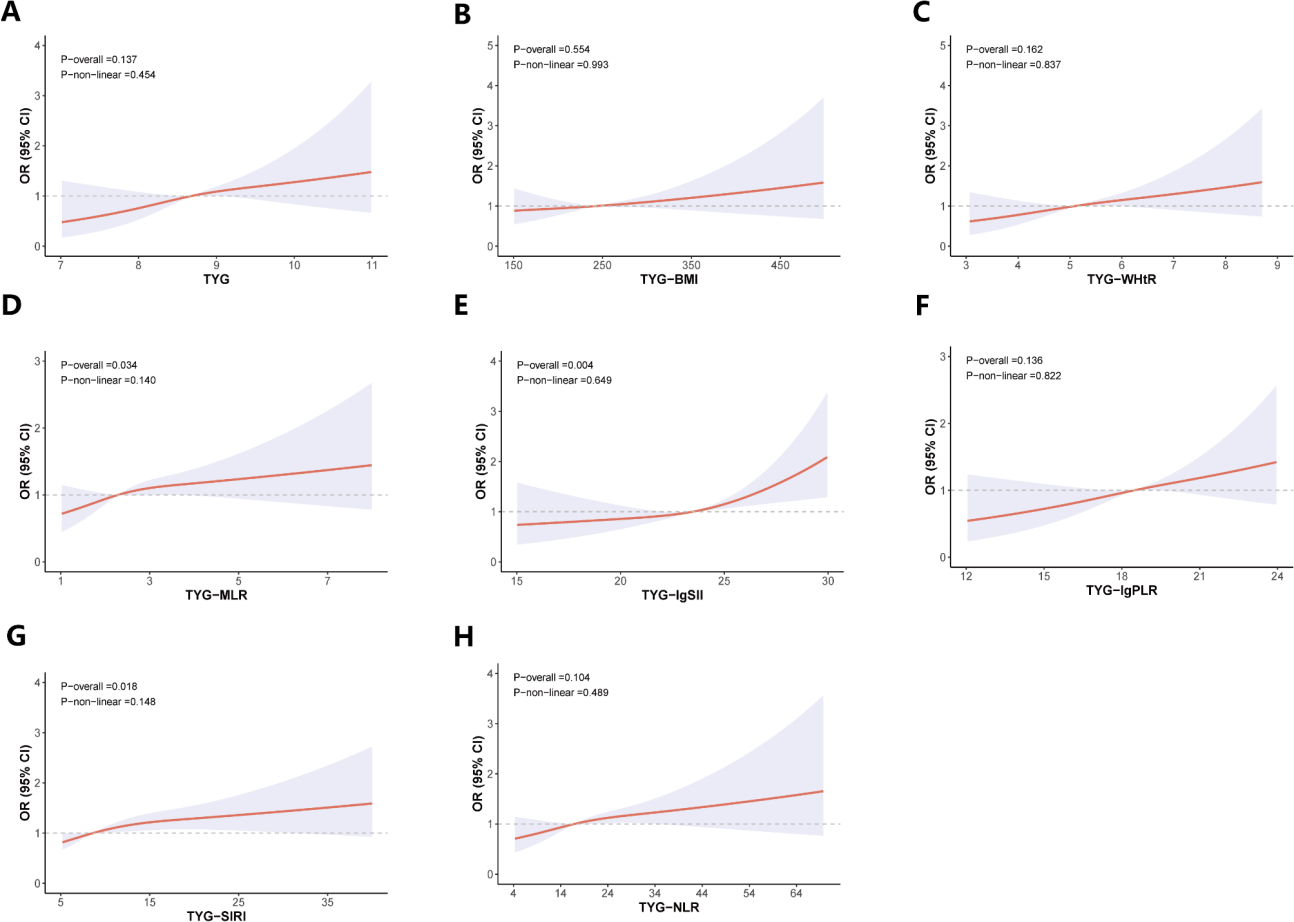
RCS analysis (**A**-**H**) demonstrated the relationship between the TyG-related index and cataract prevalence. Adjustments were made for age, gender, race, marital status, education level, income-to-poverty ratio, spouse’s education level, hypertension, alcohol use, erythrocyte count, high-density lipoprotein, and total cholesterol

Figure 2; Table 3 indicated that there was no threshold effect for TyG-SIRI, TyG-lgSII, and TyG-MLR about cataracts, showing a linear association (*P* for overall < 0.05). When TyG-SIRI and TyG-MLR were below their respective inflection points (18.23 and 3.5), their correlation with cataracts gradually increased by 5% and 23% (*P* = 0.0017; *P* = 0.0372). For TyG-lgSII values above 23, each additional unit increased the likelihood of cataracts by 12% (*P* = 0.0018).

**Table 3:** Threshold effect analysis of TyG-related index and cataract using a two-piecewise logistic regression model in adults in the NHANES 2005–2008

Threshold effect analysis	Cataract
	OR (95%CI) *P*-value
**TyG**	
Fitting by the standard linear model	1.28 (0.99, 1.67) 0.064
Inflection point of LAP (K)	9.85
< K slope	1.42 (1.05, 1.93) **0.022**
> K slope	0.64 (0.22, 1.87) 0.416
Log-likelihood ratio test	0.157
**TyG-WHtR**	
Fitting by the standard linear model	0.97 (0.86, 1.10) 0.668
Inflection point of VAI (K)	3.96
< K slope	1.91 (0.67, 5.38) 0.224
> K slope	0.93 (0.81, 1.07) 0.311
Log-likelihood ratio test	0.196
**TyG-BMI**	
Fitting by the standard linear model	1.00 (1.00, 1.00) 0.171
Inflection point of AIP (K)	185.33
< K slope	1.00 (0.98, 1.01) 0.611
> K slope	1.00 (1.00, 1.00) 0.129
Log-likelihood ratio test	0.499
**TyG-NLR**	
Fitting by the standard linear model	1.01 (1.00, 1.02) **0.026**
Inflection point of AIP (K)	27.9
< K slope	1.03 (1.00, 1.05) **0.019**
> K slope	1.00 (0.98, 1.02) 0.929
Log-likelihood ratio test	0.172
**TyG-MLR**	
Fitting by the standard linear model	1.09 (0.99, 1.20) 0.073
Inflection point of AIP (K)	3.5
< K slope	1.23 (1.01, 1.50) **0.037**
> K slope	1.01 (0.88, 1.17) 0.841
Log-likelihood ratio test	0.165
**TyG-lgPLR**	
Fitting by the standard linear model	1.08 (1.01, 1.16) **0.022**
Inflection point of AIP (K)	20.11
< K slope	1.11 (1.01, 1.21) **0.029**
> K slope	1.01 (0.84, 1.21) 0.903
Log-likelihood ratio test	0.442
**TyG-lgSII**	
Fitting by the standard linear model	1.08 (1.03, 1.14) **0.001**
Inflection point of AIP (K)	23
< K slope	1.02 (0.93, 1.13) 0.635
> K slope	1.12 (1.04, 1.20) **0.002**
Log-likelihood ratio test	0.220
**TyG-SIRI**	
Fitting by the standard linear model	1.02 (1.00, 1.04) **0.012**
Inflection point of AIP (K)	18.23
< K slope	1.05 (1.02, 1.08) **0.002**
> K slope	1.00 (0.97, 1.02) 0.864
Log-likelihood ratio test	0.340

age, gender, race, marital status, education level, income-to-poverty ratio, person’s spouse education level, hypertension, alcohol, erythrocyte, high-density lipoprotein, and total cholesterol were adjusted. Significant values are in [bold]. Non-linear relationships, including threshold and saturation effects, were explored using generalized additive models (GAMs). Abbreviations: OR, odds ratio; 95% CI, 95% confidence interval; TyG, triglyceride-glucose index; WHtR, body mass index; BMI, Body Mass Index; NLR, Neutrophil-to-Lymphocyte Ratio; MLR, Monocyte-to-Lymphocyte Ratio; lgPLR, Log-Transformed Platelet-to-Lymphocyte Ratio; lgSII, Log-Transformed Systemic Immune-Inflammation Index; SIRI, Systemic Inflammation Response Index

### ROC analysis

Among all eight TyG-related indices, TyG-MLR demonstrated the highest discriminatory ability (AUC = 0.6767; *P* < 0.001) (Table 4).

**Table 4:** ROC curves of TyG-related index for cataract

Variable	Sensitivity	Specificity	AUC (95%CI)	*P*-value	Standard error
TyG	0.754	0.385	0.584(0.557, 0.611)	< 0.001	0.073
TyG-WHtR	0.703	0.470	0.603(0.577, 0.630)	< 0.001	0.048
TyG-BMI	0.839	0.207	0.505(0.477, 0.532)	0.900	< 0.001
TyG-NLR	0.501	0.706	0.624(0.595, 0.653)	< 0.001	0.004
TyG-MLR	0.628	0.652	0.677(0.649, 0.704)	< 0.001	0.040
TyG-lgPLR	0.382	0.766	0.591(0.562, 0.620)	< 0.001	0.027
TyG-lgSII	0.708	0.472	0.598(0.569, 0.626)	< 0.001	0.018
TyG-SIRI	0.584	0.660	0.647(0.619, 0.674)	< 0.001	0.005

Abbreviations: AUC, area under curve; ROC, receiver operating characteristics curve; 95% CI, 95% confidence interval; TyG, triglyceride-glucose index; WHtR, body mass index; BMI, Body Mass Index; NLR, Neutrophil-to-Lymphocyte Ratio; MLR, Monocyte-to-Lymphocyte Ratio; lgPLR, Log-Transformed Platelet-to-Lymphocyte Ratio; lgSII, Log-Transformed Systemic Immune-Inflammation Index; SIRI, Systemic Inflammation Response Index

### Subgroup analyses

Table S5 presents the detailed subgroup analysis. Among drinking status, TyG-related inflammatory factors (NLR, MLR, lgPLR, lgSII, and SIRI) were significantly associated with an increased risk of cataracts (*P* < 0.05). However, this association was not observed in other categorical variables.

## Discussion

This study highlights a significant positive correlation between various TyG-related indices, particularly those incorporating inflammatory markers, and the prevalence of cataracts. Notably, indices that capture both metabolic and inflammatory states, such as TyG-WHtR, TyG-NLR, TyG-lgPLR, and TyG-lgSII, demonstrate a stronger predictive association with cataracts. These findings suggest that the interaction between metabolic dysfunction and chronic inflammation may play a critical role in cataract formation [16–18]. To the best of our knowledge, this is one of the first studies to systematically evaluate the relationship between TyG indices and cataract risk in a large, age-stratified population. These findings may provide novel evidence linking metabolic-inflammatory interactions to cataractogenesis.

The TyG-related indices effectively reflect insulin resistance and inflammatory states. Insulin resistance is closely associated with increased oxidative stress, which has been established as a key factor in lens protein degeneration and cataract formation [19–21]. Specifically, insulin resistance raises the oxidative burden in the body, leading to the accumulation of free radicals in the lens, impairing its transparency, and promoting lens opacification [22–25]. Indices like TyG-NLR and TyG-MLR, which incorporate inflammatory markers, effectively represent systemic inflammation. In this study, indices with inflammation-related components demonstrated stronger predictive capabilities, potentially because chronic inflammation exacerbates oxidative stress in the lens, accelerates protein aggregation, and thereby promotes cataract progression [26, 27]. These mechanisms suggest that metabolic disorders and inflammatory responses may jointly contribute to cataract formation and progression through a combined metabolic-inflammatory pathway.

Through age-stratified analysis, this study reveals the association between the TyG index and its derived indicators with cataract risk, demonstrating significant differences across various age groups. In the 20–40 age group, the TyG index and most of its derivatives showed no significant correlation, likely due to the lower burden of metabolic disorders and chronic inflammation, as well as the generally low cataract incidence in this cohort. However, in the 40–60 age group, indicators such as TyG-NLR and TyG-MLR were significantly associated with cataract risk, suggesting that metabolic and inflammatory factors begin to play an important role in cataract formation during middle age. In the 61–85 age group, these associations were further amplified, with most TyG-derived indicators (such as TyG-lgPLR, TyG-lgSII, and TyG-SIRI) remaining significantly correlated with cataract risk in fully adjusted models, exhibiting higher odds ratios. This suggests that the cumulative effects of metabolic dysfunction and chronic low-grade inflammation are pivotal in cataract onset and progression among the elderly.

These findings validate the role of metabolic and inflammatory factors as key determinants of cataract risk. They also emphasize the importance of personalized interventions across different age groups. In younger populations, combining genetic and environmental factors is essential to improving predictive accuracy. In middle-aged and older populations, incorporating TyG-related indicators into routine cataract screening strategies, along with strengthening metabolic control, and anti-inflammatory, and antioxidant treatments, could help reduce cataract incidence in high-risk individuals. Future research should further explore the specific associations between TyG-related indicators and different cataract subtypes (such as nuclear, cortical, and posterior subcapsular cataracts) while integrating longitudinal studies and interventional trials to elucidate the dynamic effects of metabolic-inflammatory interactions in cataract pathogenesis.

RCS analysis further revealed that TyG-SIRI, TyG-lgSII, and TyG-MLR are continuously and linearly associated with cataract prevalence, yet no significant threshold effect was observed. This finding suggests that even moderate increases in metabolic and inflammatory markers may elevate cataract risk, potentially reflecting the cumulative impact of metabolic and inflammatory stress on lens protein damage. The linear increase in risk highlights the importance of early intervention for metabolic abnormalities and inflammation, emphasizing the role of metabolic health in cataract prevention. The absence of a threshold effect may indicate that cataract progression is related to the cumulative burden of metabolic and inflammatory stress rather than being triggered by a single event or critical threshold. Consistent with similar findings on the TyG index in other chronic diseases (such as cardiovascular disease and diabetes), this study further validates the widespread impact of cumulative metabolic and chronic inflammatory stress on chronic disease risk.

This study shows that TyG-MLR and TyG-SIRI perform well in predicting cataract risk, remaining significant even after adjusting for other variables. These indices serve as combined metabolic-inflammatory markers, aiding clinicians in identifying individuals at higher risk for cataracts, particularly among older adults with metabolic syndrome or chronic inflammatory conditions. The TyG index and its derivatives are easily obtainable through routine clinical assessments, offering good feasibility and broad applicability. Although surgery is currently the only definitive treatment for cataracts, using TyG-related indices for early identification of high-risk individuals could facilitate early interventions or personalized health management, potentially reducing cataract incidence or delaying its progression. Specifically, for individuals with a higher metabolic and inflammatory burden, interventions might include intensified glucose and lipid control, antioxidant intake, and anti-inflammatory treatments, which may help slow cataract development [28, 29].

In recent years, the TyG index has garnered significant attention as a reliable indicator of insulin resistance and metabolic dysfunction. An increasing body of research has explored its association with various chronic conditions, including diabetes, cardiovascular diseases, and even cataract formation. A study by Tian et al. (2024) revealed a significant correlation between the TyG index and the risk of aortic dissection and aneurysm, underscoring its potential as an early marker for life-threatening cardiovascular diseases [30]. Similarly, in diabetes, an investigation of the TyG index about new-onset diabetes in the National Longitudinal Study suggested that higher TyG values are associated with an increased risk of developing diabetes [31]. These findings highlight the crucial role that the TyG index may play in the pathogenesis of metabolic diseases, with potential implications for age-related conditions such as cataracts. Recent studies have increasingly highlighted the central role of oxidative stress and chronic inflammation in the pathogenesis of various types of cataracts, not limited to age-related cataracts (ARC), but also including diabetic, hereditary, and nuclear cataracts. A recent review by Hejtmancik indicates that, despite the limited number of identified antioxidant genes, these genes still provide valuable clues for scientists investigating the antioxidant mechanisms in the lens [32]. Similarly, Liu et al. identified four key genes, suggesting functional interactions between oxidative stress and ferroptosis genes in diabetic cataracts [33–35].

Compared to the extensive application of the TyG index in studies on chronic diseases like cardiovascular disease and diabetes, its use in ophthalmology remains in the exploratory phase. Previous research has demonstrated the significant predictive value of the TyG index for cardiovascular disease and diabetes risk [9, 36–38]. The exploration of the association between TyG-related indices and cataracts offers new perspectives for cataract prediction and early prevention. Unlike traditional cataract risk factors, the TyG index and its derivatives have the advantage of quantifying metabolic and inflammatory burdens. They provide not only a more comprehensive risk assessment but are also straightforward to implement and interpret, with the potential for clinical translation into screening tools. As the aging population grows and cataract incidence rises, screening and management strategies based on metabolic and inflammatory factors will become a critical area in preventive ophthalmology.

Therefore, TyG-related indices may serve as practical and accessible biomarkers for identifying individuals at higher risk of cataracts. Given that cataract surgery remains the only definitive treatment, identifying modifiable risk factors is essential for reducing cataract incidence and progression.

### Study strengths and limitations

This study conducted a comprehensive analysis of various TyG-related indicators to thoroughly assess the combined impact of metabolic and inflammatory biomarkers on cataract risk. Additionally, the use of multiple TyG-derived indices offers deeper insight into these associations, which could aid in identifying specific indices as practical biomarkers for cataract screening and prevention. However, this study only reveals associations. Future longitudinal studies could observe how changes in the TyG index affect cataract progression, providing a clearer understanding of their temporal relationship. Second, Cataract data based on self-reports may lead to misclassification or bias; clinical examinations or imaging assessments could yield more accurate cataract diagnoses in future studies. Additionally, this study did not consider lifestyle factors (such as diet and exercise) or genetic influences, which may be closely linked to metabolic and inflammatory states. Future research could integrate lifestyle and genetic data for a more comprehensive evaluation of the impact of TyG-related indices on cataract risk. Although this study is based on NHANES data, the relatively small sample size may limit the statistical power of the results and their generalizability, especially in subgroup analyses where sample size may be insufficient. Further interventional studies could also explore whether metabolic and inflammation management can effectively reduce cataract risk, thereby validating the application value of the TyG index in cataract prevention.

## Conclusion

In conclusion, this study underscores the relevance of TyG-related indices in clarifying the impact of metabolic and inflammatory factors on cataract development. These indices hold promise as valuable biomarkers for early detection and prevention strategies, offering new perspectives for managing cataract risk in middle-aged and elderly individuals.

## Electronic supplementary material

Below is the link to the electronic supplementary material.

Supplementary Material 1

## Data Availability

No datasets were generated or analysed during the current study.

## Abbreviations

NHANES: National health and nutrition examination survey
ROC: Receiver operating characteristic
RCS: Restricted cubic spline
TyG: Triglyceride-glucose
WHtR: Waist-to-height ratio
NLR: Neutrophil-to-lymphocyte ratio
MLR: Monocyte-to-lymphocyte ratio
PLR: Platelet-to-lymphocyte ratio
SII: Systemic inflammation index
SIRI: Systemic inflammation response index
HDL-C: High-density lipoprotein cholesterol
PIR: Poverty-income ratio
BMI: Body mass index
WC: Waist circumference
TG: Triglycerides
TC: Total cholesterol
OR: Odds ratio
CI: Confidence interval
