# Supplementary material for: Association of triglyceride-glucose index and derived indices with cataract in middle-aged and elderly Americans: NHANES 2005–2008
Source: Lipids Health Dis. 2025 Feb 14;24:48. doi: 10.1186/s12944-025-02470-4 (PMC11827319; doi:10.1186/s12944-025-02470-4)
Supplement: Supplementary file 1 — Supplementary Material 1 [file 12944_2025_2470_MOESM1_ESM.docx]

### **Table S5**

Stratified analysis of the correlation between TyG-related index and cataract in adults in the NHANES 2005–2008

| **Subgroup** | **OR(95%CI)**  **,*P*-value** | ***P* interaction** | **OR(95%CI)**  **,*P*-value** | ***P* interaction** | **OR(95%CI)**  **,*P*-value** | ***P* interaction** | **OR(95%CI)**  **,*P*-value** | ***P* interaction** | **OR(95%CI)**  **,*P*-value** | ***P* interaction** | **OR(95%CI)**  **,*P*-value** | ***P* interaction** | **OR(95%CI)**  **,*P*-value** | ***P* interaction** | **OR(95%CI)**  **,*P*-value** | ***P* interaction** |
| --- | --- | --- | --- | --- | --- | --- | --- | --- | --- | --- | --- | --- | --- | --- | --- | --- |
|  | **TyG** |  | **TyG-WHtR** |  | **TyG-BMI** |  | **TyG-NLR** |  | **TyG-MLR** |  | **TyG-lgPLR** |  | **TyG-lgSII** |  | **TyG-SIRI** |  |
| **Gender** |  | 0.346 |  | 0.274 |  | 0.400 |  | 0.231 |  | 0.785 |  | 0.774 |  | 0.857 |  | 0.624 |
| Male | 1.36 (0.95, 1.94) 0.0925 |  | 1.30 (1.02, 1.65) 0.0354 |  | 1.00 (1.00, 1.01) 0.1427 |  | 1.01 (0.99, 1.02) 0.4120 |  | 1.07 (0.95, 1.21) 0.2724 |  | 1.07 (0.97, 1.17) 0.1742 |  | 1.08 (1.01, 1.15) 0.0296 |  | 1.02 (1.00, 1.04) 0.1069 |  |
| Female | 1.07 (0.73, 1.56) 0.7443 |  | 1.08 (0.88, 1.34) 0.4568 |  | 1.00 (1.00, 1.00) 0.6549 |  | 1.02 (1.00, 1.04) 0.0197 |  | 1.10 (0.94, 1.30) 0.2378 |  | 1.09 (0.99, 1.20) 0.0899 |  | 1.09 (1.01, 1.17) 0.0174 |  | 1.03 (1.00, 1.05) 0.0562 |  |
| **Age** |  | 0.120 |  | 0.629 |  | 0.377 |  | 0.579 |  | 0.640 |  | 0.492 |  | 0.108 |  | 0.697 |
| ＜40 | 15.33 (0.91, 259.42) 0.0585 |  | 1.43 (0.59, 3.49) 0.4309 |  | 1.01 (1.00, 1.02) 0.2151 |  | 1.07 (0.96, 1.19) 0.2019 |  | 1.04 (0.40, 2.72) 0.9406 |  | 1.55 (0.80, 2.98) 0.1908 |  | 1.79 (1.04, 3.09) 0.0355 |  | 1.04 (0.94, 1.14) 0.4633 |  |
| 41-60 | 1.14 (0.54, 2.40) 0.7281 |  | 0.92 (0.56, 1.50) 0.7273 |  | 1.00 (0.99, 1.00) 0.4970 |  | 1.03 (1.00, 1.06) 0.0901 |  | 1.42 (0.96, 2.10) 0.0764 |  | 1.02 (0.80, 1.31) 0.8590 |  | 1.10 (0.93, 1.30) 0.2869 |  | 1.05 (1.00, 1.10) 0.0306 |  |
| ＞61 | 1.22 (0.93, 1.59) 0.1467 |  | 1.12 (0.96, 1.32) 0.1456 |  | 1.00 (1.00, 1.00) 0.5334 |  | 1.02 (1.01, 1.03) 0.0046 |  | 1.18 (1.07, 1.30) 0.0011 |  | 1.08 (1.01, 1.15) 0.0273 |  | 1.08 (1.03, 1.14) 0.0014 |  | 1.03 (1.01, 1.05) 0.0003 |  |
| **Race** |  | 0.988 |  | 0.670 |  | 0.610 |  | 0.054 |  | 0.394 |  | 0.416 |  | 0.069 |  | 0.121 |
| Mexican American | 1.20 (0.52, 2.77) 0.6660 |  | 1.11 (0.64, 1.92) 0.7233 |  | 1.00 (0.99, 1.01) 0.9110 |  | 0.98 (0.92, 1.04) 0.5178 |  | 1.14 (0.75, 1.74) 0.5398 |  | 0.91 (0.72, 1.15) 0.4321 |  | 0.98 (0.83, 1.16) 0.7974 |  | 1.04 (0.97, 1.12) 0.2692 |  |
| Other Hispanic | 1.19 (0.31, 4.54) 0.7948 |  | 0.89 (0.38, 2.06) 0.7792 |  | 1.00 (0.99, 1.01) 0.8074 |  | 1.09 (1.02, 1.17) 0.0156 |  | 1.97 (1.05, 3.69) 0.0359 |  | 1.39 (0.94, 2.07) 0.0997 |  | 1.47 (1.08, 2.00) 0.0132 |  | 1.13 (1.02, 1.25) 0.0164 |  |
| Non-Hispanic White | 1.42 (1.01, 2.00) 0.0417 |  | 1.17 (0.97, 1.42) 0.1074 |  | 1.00 (1.00, 1.00) 0.5132 |  | 1.02 (1.00, 1.03) 0.0185 |  | 1.05 (0.94, 1.18) 0.4054 |  | 1.10 (1.01, 1.19) 0.0260 |  | 1.11 (1.05, 1.19) 0.0006 |  | 1.02 (1.00, 1.04) 0.0355 |  |
| Non-Hispanic Black | 1.19 (0.60, 2.36) 0.6122 |  | 1.57 (1.05, 2.36) 0.0286 |  | 1.01 (1.00, 1.01) 0.0354 |  | 0.98 (0.95, 1.02) 0.3027 |  | 1.11 (0.78, 1.57) 0.5737 |  | 1.05 (0.88, 1.26) 0.5758 |  | 0.99 (0.88, 1.11) 0.8694 |  | 0.98 (0.93, 1.03) 0.4109 |  |
| Other Race | 1.85 (0.52, 6.60) 0.3424 |  | 1.13 (0.43, 2.95) 0.8041 |  | 1.00 (0.98, 1.02) 0.8295 |  | 1.01 (0.93, 1.10) 0.8607 |  | 1.68 (0.68, 4.14) 0.2584 |  | 1.29 (0.81, 2.05) 0.2862 |  | 1.13 (0.85, 1.49) 0.4039 |  | 1.05 (0.95, 1.16) 0.3646 |  |
| **Education Level** |  | 0.880 |  | 0.133 |  | 0.428 |  | 0.454 |  | 0.435 |  | 0.373 |  | 0.488 |  | 0.455 |
| Less Than 9th Grade | 1.09 (0.62, 1.92) 0.7756 |  | 1.03 (0.73, 1.46) 0.8487 |  | 1.00 (0.99, 1.01) 0.8804 |  | 1.00 (0.98, 1.03) 0.7723 |  | 1.21 (0.91, 1.61) 0.1807 |  | 1.07 (0.93, 1.25) 0.3423 |  | 1.06 (0.95, 1.19) 0.2786 |  | 1.02 (0.98, 1.06) 0.3541 |  |
| 9-11th Grade | 1.51 (0.85, 2.67) 0.1612 |  | 1.78 (1.27, 2.50) 0.0008 |  | 1.01 (1.00, 1.01) 0.0177 |  | 1.02 (1.00, 1.05) 0.0924 |  | 1.10 (0.86, 1.40) 0.4650 |  | 0.98 (0.83, 1.17) 0.8430 |  | 1.13 (1.00, 1.28) 0.0523 |  | 1.04 (1.00, 1.08) 0.0287 |  |
| High School Grad | 1.68 (0.92, 3.06) 0.0919 |  | 1.12 (0.81, 1.55) 0.4910 |  | 1.00 (0.99, 1.00) 0.8941 |  | 1.00 (0.98, 1.02) 0.9120 |  | 0.93 (0.75, 1.15) 0.5126 |  | 1.05 (0.91, 1.20) 0.5188 |  | 1.06 (0.96, 1.18) 0.2197 |  | 1.00 (0.97, 1.04) 0.7943 |  |
| Some College or AA degree | 1.37 (0.74, 2.55) 0.3210 |  | 1.15 (0.79, 1.68) 0.4671 |  | 1.00 (1.00, 1.01) 0.4556 |  | 1.03 (1.00, 1.06) 0.0733 |  | 1.28 (0.95, 1.71) 0.1027 |  | 1.25 (1.05, 1.48) 0.0114 |  | 1.17 (1.04, 1.32) 0.0099 |  | 1.04 (0.99, 1.09) 0.0874 |  |
| College Graduate or above | 1.39 (0.58, 3.32) 0.4555 |  | 0.94 (0.54, 1.63) 0.8298 |  | 1.00 (0.99, 1.01) 0.9777 |  | 1.00 (0.96, 1.03) 0.8456 |  | 1.06 (0.87, 1.30) 0.5642 |  | 1.05 (0.86, 1.28) 0.6464 |  | 1.01 (0.88, 1.15) 0.9246 |  | 1.00 (0.97, 1.04) 0.8709 |  |
| **Person's Spouse Education Level** |  | 0.751 |  | 0.118 |  | 0.296 |  | 0.094 |  | 0.057 |  | 0.008 |  | 0.144 |  | 0.535 |
| Less Than 9th Grade | 1.04 (0.39, 2.74) 0.9351 |  | 1.17 (0.65, 2.10) 0.6004 |  | 1.00 (0.99, 1.01) 0.5471 |  | 1.03 (0.98, 1.08) 0.2513 |  | 1.82 (1.11, 3.00) 0.0181 |  | 1.60 (1.12, 2.28) 0.0092 |  | 1.22 (0.97, 1.52) 0.0829 |  | 1.04 (0.97, 1.10) 0.2745 |  |
| 9-11th Grade | 2.38 (0.81, 7.05) 0.1168 |  | 2.50 (1.20, 5.20) 0.0141 |  | 1.01 (1.00, 1.02) 0.0290 |  | 0.99 (0.93, 1.05) 0.6993 |  | 0.90 (0.50, 1.63) 0.7281 |  | 0.98 (0.70, 1.37) 0.9123 |  | 1.10 (0.87, 1.39) 0.4420 |  | 1.01 (0.93, 1.10) 0.7792 |  |
| High School Grad | 1.54 (0.72, 3.30) 0.2696 |  | 1.58 (1.01, 2.48) 0.0446 |  | 1.00 (1.00, 1.01) 0.3985 |  | 1.01 (0.98, 1.05) 0.4285 |  | 0.97 (0.73, 1.30) 0.8579 |  | 1.11 (0.92, 1.34) 0.2558 |  | 1.16 (1.01, 1.33) 0.0406 |  | 1.03 (0.98, 1.08) 0.2203 |  |
| Some College or AA degree | 1.81 (0.78, 4.21) 0.1691 |  | 1.40 (0.79, 2.49) 0.2532 |  | 1.01 (1.00, 1.01) 0.2661 |  | 1.07 (1.02, 1.12) 0.0044 |  | 1.71 (1.14, 2.56) 0.0089 |  | 1.54 (1.17, 2.02) 0.0019 |  | 1.30 (1.09, 1.54) 0.0028 |  | 1.08 (1.02, 1.15) 0.0145 |  |
| College Graduate or above | 1.39 (0.46, 4.23) 0.5644 |  | 1.23 (0.65, 2.34) 0.5314 |  | 1.00 (0.99, 1.01) 0.5392 |  | 0.97 (0.92, 1.02) 0.2599 |  | 0.93 (0.60, 1.45) 0.7598 |  | 0.87 (0.65, 1.17) 0.3568 |  | 0.96 (0.78, 1.18) 0.6804 |  | 0.99 (0.93, 1.07) 0.8881 |  |
| Other | 1.14 (0.79, 1.65) 0.4844 |  | 1.00 (0.81, 1.24) 0.9785 |  | 1.00 (1.00, 1.00) 0.8100 |  | 1.01 (1.00, 1.03) 0.0949 |  | 1.07 (0.95, 1.21) 0.2786 |  | 1.04 (0.95, 1.14) 0.4454 |  | 1.06 (0.99, 1.13) 0.1016 |  | 1.02 (1.00, 1.04) 0.0728 |  |
| **Marital status** |  | 0.063 |  | 0.904 |  | 0.739 |  | 0.473 |  | 0.876 |  | 0.873 |  | 0.987 |  | 0.529 |
| Married | 1.38 (0.95, 2.00) 0.0895 |  | 1.30 (1.04, 1.64) 0.0228 |  | 1.00 (1.00, 1.01) 0.0527 |  | 1.01 (1.00, 1.03) 0.1396 |  | 1.13 (0.96, 1.32) 0.1470 |  | 1.11 (1.00, 1.22) 0.0428 |  | 1.10 (1.03, 1.18) 0.0065 |  | 1.02 (1.00, 1.05) 0.0501 |  |
| Widowed | 1.28 (0.73, 2.26) 0.3888 |  | 1.06 (0.78, 1.44) 0.6974 |  | 1.00 (0.99, 1.00) 0.7979 |  | 1.01 (0.99, 1.03) 0.4355 |  | 1.08 (0.90, 1.30) 0.4178 |  | 1.09 (0.96, 1.23) 0.1943 |  | 1.07 (0.97, 1.17) 0.1617 |  | 1.01 (0.98, 1.04) 0.6079 |  |
| Divorced | 1.68 (0.80, 3.50) 0.1689 |  | 1.21 (0.79, 1.88) 0.3828 |  | 1.00 (1.00, 1.01) 0.5760 |  | 1.03 (0.99, 1.08) 0.1040 |  | 0.97 (0.68, 1.39) 0.8787 |  | 1.04 (0.84, 1.29) 0.7005 |  | 1.14 (0.98, 1.34) 0.0878 |  | 1.06 (1.00, 1.12) 0.0604 |  |
| Separated | 0.61 (0.12, 3.03) 0.5473 |  | 1.65 (0.62, 4.36) 0.3136 |  | 1.00 (0.99, 1.02) 0.5337 |  | 28.33 (0.00, inf.) 0.9846 |  | 1.28 (0.80, 2.04) 0.3009 |  | 0.97 (0.59, 1.61) 0.9184 |  | 1.01 (0.99, 1.03) 0.3077 |  | 1.05 (0.96, 1.14) 0.2792 |  |
| Never married | 0.03 (0.00, 1.05) 0.0531 |  | 2.07 (0.42, 10.26) 0.3737 |  | 1.01 (0.99, 1.03) 0.2352 |  | 1.11 (0.99, 1.25) 0.0853 |  | 1.18 (0.47, 2.99) 0.7254 |  | 0.70 (0.34, 1.47) 0.3484 |  | 1.13 (0.77, 1.67) 0.5374 |  | 1.10 (0.94, 1.28) 0.2200 |  |
| Living with partner | 8.09 (1.09, 60.33) 0.0413 |  | 1.36 (0.46, 4.04) 0.5779 |  | 1.00 (0.99, 1.02) 0.7970 |  | 0.99 (0.94, 1.05) 0.7200 |  | 2.02 (0.65, 6.26) 0.2216 |  | 1.02 (0.61, 1.72) 0.9281 |  | 1.12 (0.84, 1.49) 0.4537 |  | 1.09 (0.98, 1.22) 0.1211 |  |
| **Smoke** |  | 0.558 |  | 0.472 |  | 0.746 |  | 0.872 |  | 0.458 |  | 0.686 |  | 0.928 |  | 0.857 |
| YES | 1.19 (0.85, 1.66) 0.3034 |  | 1.12 (0.91, 1.38) 0.2884 |  | 1.00 (1.00, 1.00) 0.4170 |  | 1.01 (1.00, 1.03) 0.0615 |  | 1.07 (0.95, 1.21) 0.2610 |  | 1.07 (0.98, 1.17) 0.1441 |  | 1.09 (1.02, 1.16) 0.0113 |  | 1.02 (1.00, 1.04) 0.0305 |  |
| NO | 1.39 (0.92, 2.08) 0.1169 |  | 1.26 (0.99, 1.59) 0.0592 |  | 1.00 (1.00, 1.01) 0.2467 |  | 1.01 (0.99, 1.03) 0.2104 |  | 1.16 (0.99, 1.36) 0.0754 |  | 2.51 (0.52, 12.19) 0.2537 |  | 1.08 (1.00, 1.17) 0.0427 |  | 1.02 (0.99, 1.05) 0.1470 |  |
| **Alcohol drinking** |  | 0.037 |  | 0.176 |  | 0.239 |  | <0.001 |  | 0.023 |  | <0.001 |  | <0.001 |  | <0.001 |
| YES | 1.18 (0.83, 1.66) 0.3594 |  | 1.10 (0.90, 1.35) 0.3468 |  | 1.00 (1.00, 1.00) 0.8944 |  | 1.02 (1.00, 1.03) 0.0109 |  | 1.14 (1.01, 1.29) 0.0413 |  | 1.14 (1.04, 1.25) 0.0051 |  | 1.11 (1.04, 1.18) 0.0014 |  | 1.02 (1.00, 1.05) 0.0170 |  |
| NO | 1.43 (0.90, 2.28) 0.1282 |  | 1.26 (0.97, 1.62) 0.0801 |  | 1.00 (1.00, 1.01) 0.1634 |  | 0.99 (0.97, 1.01) 0.4026 |  | 0.95 (0.80, 1.13) 0.5578 |  | 0.98 (0.87, 1.09) 0.6998 |  | 1.02 (0.94, 1.10) 0.7019 |  | 1.00 (0.97, 1.03) 0.8266 |  |
| Other | 1.50 (0.54, 4.17) 0.4386 |  | 1.23 (0.58, 2.60) 0.5876 |  | 1.02 (0.99, 1.05) 0.1920 |  | 1.11 (1.04, 1.18) 0.0010 |  | 2.40 (1.04, 5.55) 0.0408 |  | 1.51 (1.00, 2.27) 0.0509 |  | 1.50 (1.10, 2.05) 0.0102 |  | 1.10 (1.04, 1.17) 0.0006 |  |
| **Hypertension** |  | 0.182 |  | 0.183 |  | 0.160 |  | 0.554 |  | 0.719 |  | 0.395 |  | 0.250 |  | 0.973 |
| YES | 1.13 (0.82, 1.58) 0.4520 |  | 1.28 (1.05, 1.56) 0.0131 |  | 1.00 (1.00, 1.01) 0.0541 |  | 1.01 (1.00, 1.03) 0.1270 |  | 1.11 (0.99, 1.25) 0.0731 |  | 1.06 (0.97, 1.16) 0.1686 |  | 1.06 (1.00, 1.13) 0.0521 |  | 1.02 (1.00, 1.04) 0.0382 |  |
| NO | 1.61 (1.07, 2.43) 0.0237 |  | 1.03 (0.80, 1.33) 0.8199 |  | 1.00 (0.99, 1.00) 0.7075 |  | 1.02 (1.00, 1.04) 0.0645 |  | 1.07 (0.90, 1.28) 0.4525 |  | 1.13 (1.01, 1.25) 0.0298 |  | 1.13 (1.04, 1.22) 0.0027 |  | 1.02 (0.99, 1.05) 0.1188 |  |
| **Diabetes** |  | 0.811 |  | 1.000 |  | 0.980 |  | 0.634 |  | 0.976 |  | 0.979 |  | 0.762 |  | 0.515 |
| YES | 0.98 (0.59, 1.62) 0.9233 |  | 1.12 (0.80, 1.57) 0.5088 |  | 1.00 (1.00, 1.01) 0.8527 |  | 1.02 (1.00, 1.05) 0.0721 |  | 1.13 (0.91, 1.41) 0.2666 |  | 1.08 (0.95, 1.22) 0.2436 |  | 1.11 (1.00, 1.22) 0.0497 |  | 1.04 (1.00, 1.08) 0.0298 |  |
| NO | 1.20 (0.84, 1.72) 0.3274 |  | 1.12 (0.92, 1.36) 0.2539 |  | 1.00 (1.00, 1.00) 0.4999 |  | 1.01 (1.00, 1.02) 0.1906 |  | 1.10 (0.98, 1.23) 0.0959 |  | 1.06 (0.98, 1.15) 0.1584 |  | 1.06 (1.00, 1.12) 0.0587 |  | 1.02 (1.00, 1.04) 0.0739 |  |
| Other | 0.46 (0.11, 1.92) 0.2867 |  | 0.46 (0.19, 1.13) 0.0914 |  | 0.99 (0.97, 1.00) 0.0800 |  | 1.02 (0.95, 1.09) 0.6092 |  | 0.97 (0.62, 1.53) 0.9090 |  | 0.92 (0.66, 1.29) 0.6413 |  | 0.98 (0.76, 1.27) 0.9009 |  | 1.00 (0.93, 1.09) 0.9089 |  |

The results show that the subgroup analysis was adjusted for all presented covariates except the effect modifier.

Stratified analysis were analyzed using generalized linear models (GLMs) with interaction terms.

Abbreviations: 95% CI, 95% confidence interval; OR, odds ratio.
